# Macular hole after Nd‐YAG laser capsulotomy with OCT findings

**DOI:** 10.1002/ccr3.4267

**Published:** 2021-05-15

**Authors:** Tsutomu Ohashi, Akio Fujiya, Takashi Kojima

**Affiliations:** ^1^ Ohashi Eye Center Sapporo Japan; ^2^ Department of Ophthalmology Keio University School of Medicine Tokyo Japan

**Keywords:** aftercataract, macular hole, Nd‐YAG laser capsulotomy, OCT

## Abstract

Patients with incomplete posterior vitreous detachment, especially with vitreomacular adhesion, can form a macular hole after Nd‐YAG laser capsulotomy. It is recommended to inform the risk of forming a macular hole before Nd‐YAG laser treatment.

## Graphical abstract

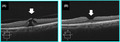

## 1 INTRODUCTION

A macular hole is a rare complication that appears after Nd‐YAG laser capsulotomy. We will present clear high‐definition optical coherence tomography (HD‐OCT) images that suggest that Nd‐YAG laser may accelerate the formation of macular holes and direct Nd‐YAG pulse wave may not influence this formation in our case.

A macular hole (MH) is a retinal defect in the macula which leads to severe visual disturbance. Vitreomacular adhesion is well recognized as a major cause of macular hole development.^1^, ^2^

However, macular hole formation is also reported as a complication that appears after neodymium‐doped yttrium aluminum garnet (Nd‐YAG) laser capsulotomy for posterior capsule opacification after cataract surgery. Nd‐YAG laser capsulotomy has been associated with complications such as retinal detachment, cystoid macular edema, raised intra‐ocular pressure (IOP), intra‐ocular lens damage, and endophthalmitis.^3^, ^4^, ^5^, ^6^ Although it is speculated that the perifoveal vitreous contraction,^7^ vitreous instability^8^ due to the vitreous liquefaction, and YAG laser wave pulse^9^ could be the causes of the macular hole formation after Nd‐YAG laser capsulotomy,^7^, ^10^ other reports claim that patients without vitreomacular adhesion after YAG laser capsulotomy showed macular hole formation,^11^, ^12^ suggesting the effects of laser wave pulse. The precise etiology remains to be explained.

A macular hole following Nd‐YAG laser capsulotomy after capsular opacification with pre‐existing vitreomacular adhesion is presented in this report. Nd‐YAG laser capsulotomy was performed bilaterally. Right eye had a complete posterior vitreous detachment before laser treatment. In contrast, left eye had incomplete posterior vitreous detachment with vitreomacular adhesion, which is speculated to cause a macular hole. Although many reports^4^, ^7^, ^8^, ^9^, ^10^, ^11^, ^12^ exist regarding macular hole after Nd‐YAG laser capsulotomy, there has been no very clear high‐definition optical coherence tomography (HD‐OCT) before and after YAG laser images which clarify the cause of the macular hole formation except for the Umut's case.^10^ In our case, HD‐OCT images very clearly show pre‐existing vitreomacular adhesion before applying Nd‐YAG laser capsulotomy and a formed full‐thickness macular hole after Nd‐YAG laser capsulotomy only in the eye with vitreomacular adhesion.

## 2 CASE PRESENTATION

**FIGURE 1:**
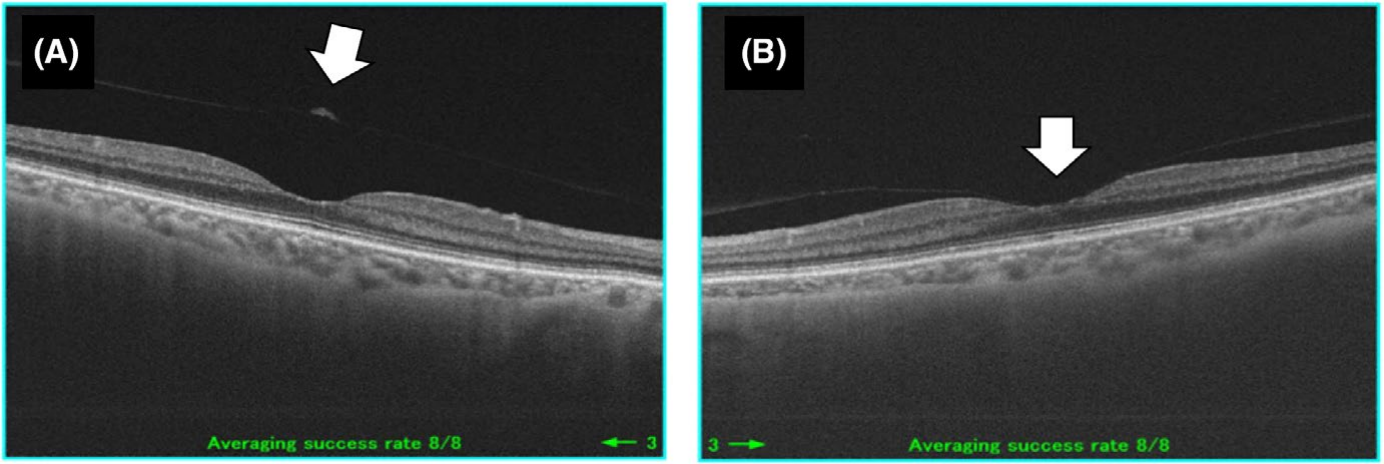
High‐definition optical coherence tomography (HD‐OCT) before applying Nd‐YAG laser for posterior capsule opacification. Complete posterior vitreous detachment was shown in the right eye (A, white arrow). In contrast, focal vitreomacular adhesion was observed in the left eye (B, white arrow)

A 67‐year‐old female with a visually significant cataract (visual acuity 20/40 in both eyes) underwent cataract surgery (HOYA Acryfold). The patient had no significant history of systemic disease. Preoperatively, the right eye had a complete posterior vitreous detachment (Figure 1A), the left eye had incomplete posterior vitreous detachment with vitreomacular adhesion (Figure 1B). Postoperatively, her visual acuity improved to 20/17.

**FIGURE 2:**
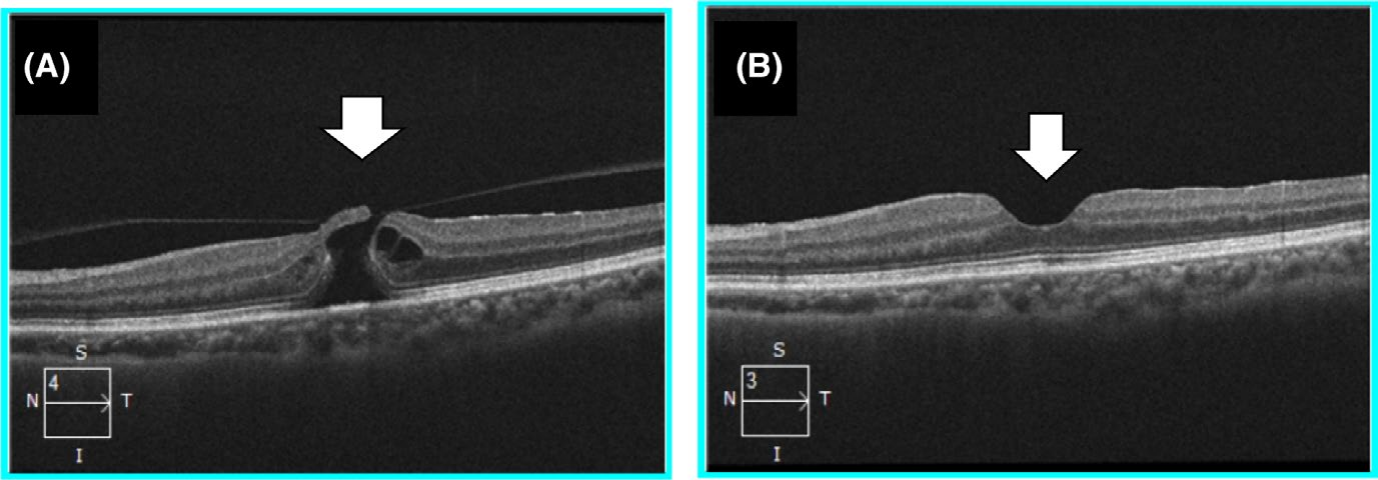
High‐definition optical coherence tomography (HD‐OCT) after applying Nd‐YAG laser for posterior capsule opacification. The presence of incomplete posterior vitreous detachment was confirmed and a full‐thickness macular hole was formed in the left eye (A, white arrow). Three months after Pars Plana Vitrectomy, HD‐OCT image showed complete closure of macular hole in the left eye (B, white arrow)

A year and four months postoperatively, Nd‐YAG laser capsulotomy was performed since bilateral capsular opacification was found in both eyes and her visual acuity had deteriorated to 20/22 in the right eye, and 20/28 in the left eye. After Nd‐YAG laser capsulotomy (1.2 mJ/pulse, total energy 25.2 mJ in both eyes) was performed, her blurred vision disappeared. However, the patient noticed a decrease in visual acuity and central distortion in the left eye two months after Nd‐YAG laser capsulotomy. Her visual acuity in the left eye was 20/100 at the time. HD‐OCT examination of her left eye showed a full‐thickness macular hole (Figure 2A). Pars plana vitrectomy (PPV) and the internal limiting membrane (ILM) peeling with gas were performed. After the surgery, her HD‐OCT showed the closure of the macular hole (Figure 2B). One month postoperatively, her visual acuity improved to 20/28.

## 3 DISCUSSION

Although Nd‐YAG laser capsulotomy was a common, safe and effective outpatient procedure after cataract surgery with rare complications, previous literature described various complications induced by Nd‐YAG laser capsulotomy. Li et al^5^ presented the percentage of complications after Nd‐YAG laser capsulotomy. In their clinical data, 497 patients received Nd‐YAG laser capsulotomy, and 48 patients had complications. Percentage is as follows: rupture of the anterior hyaloid face (31.3%), macular edema (22.9%), retinal tears (16.7%), posterior vitreous detachment (12.5%), retinal detachment (12.5%), neovascular glaucoma (6.3%) and macular hole (2.0%).

There are two speculated mechanisms for causing complications of Nd‐YAG laser capsulotomy. Several authors have reported that the transmission of the Nd‐YAG laser pulse wave can directly lead to the formation of a macular hole.^11^ However, Chaudhary et al^7^ reported that the contraction of the vitreous fiber is considered the main pathogenic theory of macular hole formation after Nd‐YAG laser capsulotomy. In our case, the visual disturbance was seen two months after Nd‐YAG laser capsulotomy. Only the eye with incomplete vitreous detachment showed a macular hole. This suggests that the contraction of the vitreous fiber after Nd‐YAG laser capsulotomy might play a role in macular hole formation, and YAG laser pulse wave itself does not cause macular hole formation. In addition, relatively low energy was used (1.2 mJ/pulse, total energy 25.2 mJ) during her surgery. This means that the transmission of the Nd‐YAG laser pulse wave might not be the cause of macular hole formation^7^ in our case.

There is also a possibility that this macular hole formation was due to the natural course of vitreomacular adhesion, but two months after Nd‐YAG laser treatment, this patient developed visual disturbance, suggesting the relationship between the Nd‐YAG laser capsulotomy and macular hole formation. Several reports including our case have shown that macular hole formation^7^, ^8^, ^9^, ^10^, ^12^ occurs a short time after YAG laser capsulotomy.

In our case, only the left eye with vitreomacular pre‐existing adhesion preoperatively formed the macular hole after Nd‐YAG laser capsulotomy, while the right eye with complete vitreous detachment preoperatively did not form a macular hole after Nd‐YAG laser capsulotomy and YAG laser wave pulse itself might not be enough strong to cause a macular hole in the right eye with complete vitreous detachment. This suggests that the pre‐existing vitreomacular adhesions played a major role in a macular hole formation after Nd‐YAG laser capsulotomy. In addition, Nd‐YAG laser capsulotomy may accelerate the course of vitreous adhesion to the macula by increasing the vitreomacular traction, resulting in the formation of a hole in the macula.

Therefore, if there is an adhesion of vitreous to the macula, it is recommended to inform the patient about the risk of a macular hole forming before Nd‐YAG laser treatment.

## CONFLICT OF INTEREST

Dr Kojima declares personal fees from Staar Surgical, personal fees from Santen Pharmaceutical, personal fees from Otsuka Pharmaceutical, personal fees from Johnson & Johnson, personal fees from Alcon, outside the submitted work.

## AUTHOR CONTRIBUTIONS

All authors contributed to the idea, conceptual advice, and framework of the manuscript. AF performed the surgery, TO wrote the manuscript, TK reviewed and revised the manuscript. All authors read and approved the final manuscript.

## ETHICS APPROVAL AND CONSENT TO PARTICIPATE

This research complied with the guidelines for human studies and was conducted ethically in accordance with the World Medical Association Declaration of Helsinki. Written informed consent was obtained from the patient.

## CONSENT FOR PUBLICATION

The patient gave written informed consent to the publication of this case report (including publication of images).

## Data Availability

The data that support the findings of this study are available from the corresponding author upon reasonable request.
